# Hierarchical non-negative matrix factorization using clinical information for microbial communities

**DOI:** 10.1186/s12864-021-07401-y

**Published:** 2021-02-04

**Authors:** Ko Abe, Masaaki Hirayama, Kinji Ohno, Teppei Shimamura

**Affiliations:** 1 Division of Systems Biology, Nagoya University Graduate School of Medicine, 65 Tsurumai-cho, Showa-ku, Nagoya, 4668550 Japan; 2 School of Health Sciences, Nagoya University Graduate School of Medicine, 1-1-20 Daiko-Minami, Higashi-ku, Nagoya, 61-8873 Japan; 3 Division of Neurogenetics, Center for Neurological Diseases and Cancer, Nagoya University Graduate School of Medicine, 65 Tsurumai-cho, Showa-ku, Nagoya, 4668550 Japan; 4 Division of Systems Biology, Nagoya university Graduate School of Medicine, 65 Tsurumai-cho, Showa-ku, Nagoya, 4668550 Japan

**Keywords:** Metagenomics, Non-negative matrix factorization, Bayesian hierarchical modeling

## Abstract

**Background:**

The human microbiome forms very complex communities that consist of hundreds to thousands of different microorganisms that not only affect the host, but also participate in disease processes. Several state-of-the-art methods have been proposed for learning the structure of microbial communities and to investigate the relationship between microorganisms and host environmental factors. However, these methods were mainly designed to model and analyze single microbial communities that do not interact with or depend on other communities. Such methods therefore cannot comprehend the properties between interdependent systems in communities that affect host behavior and disease processes.

**Results:**

We introduce a novel hierarchical Bayesian framework, called BALSAMICO (BAyesian Latent Semantic Analysis of MIcrobial COmmunities), which uses microbial metagenome data to discover the underlying microbial community structures and the associations between microbiota and their environmental factors. BALSAMICO models mixtures of communities in the framework of nonnegative matrix factorization, taking into account environmental factors. We proposes an efficient procedure for estimating parameters. A simulation then evaluates the accuracy of the estimated parameters. Finally, the method is used to analyze clinical data. In this analysis, we successfully detected bacteria related to colorectal cancer.

**Conclusions:**

These results show that the method not only accurately estimates the parameters needed to analyze the connections between communities of microbiota and their environments, but also allows for the effective detection of these communities in real-world circumstances.

**Supplementary Information:**

The online version contains supplementary material available at (10.1186/s12864-021-07401-y).

## Background

Microbiota in the human gut form complex communities that consist of hundreds to thousands of different microorganisms that affect various important functions such as the maturation of the immune system, physiology [1], metabolism [2], and nutrient circulation [3]. Species in a community survive by interacting with each other and can concurrently belong to multiple communities [4]. Moreover, the composition of bacterial species can change over time. In some cases, a single species or strain significantly affects the state of the community, making it a causative agent for disease. For example, *Helicobacter pylori* is a pathogen that induces peptic disease [5]. However, problems are not always rooted in an individual species or strain. In many cases it is the differences in different types of microbial communities, i.e. their composition ratios, that affect the overall structure of the gut microbiota. These overall structures relate to various features of interest— for example, the ecosystem process [6], the severity of the disease [7], or the impact of dietary intervention [8]. Therefore, finding co-occurrence relationships between species and revealing the community structure of microorganisms is crucial to understanding the principles and mechanisms of microbiota-associated health and disease relationships and interactions between the host and microbe.

Thanks to modern technology, revealing these community structures is becoming easier. Advances in high-throughput sequencing technologies such as shotgun metagenomics have made it possible to investigate the relationship among microorganisms within the whole gut ecosystem and to observe the interaction between microbiota and their host environments. Many microbiome projects, including the Human Microbiome Project (HMP) [9] and the Metagenomics and the Human Intestinal Tract (MetaHIT) project [10], have generated considerable data regarding human microbiota by studying microbial diversity in different environments. The data consists of either marker-gene data (the abundance of operational taxonomic units; OTUs) or functional metagenomic data (the abundance of reaction-coding enzymes). Although collecting such data is no longer methodologically difficult, analysis remains challenging. Even with limited samples, the data always consists of hundreds or even thousands of variables (OTUs or enzymes). In addition, there are many rare species of microbiota, and these are observed only in very few samples. Thus the data is highly sparse [11]. The sparse nature of the data means that classical statistical analysis methods, which were designed for data rich situations, have limited ability to identify complex features and structures within the data. Several new methods are therefore emerging in order to properly analyze and understand microbiota.

A main challenge in metagenomic data analysis is to learn the structure of microbial communities and to investigate the relationship between microorganisms and their environmental factors. Currently, there are several methods that seek to clarify this relationship. One is probabilistic modeling of metagenomic data, which often provides a powerful framework for the problem. For example, [13] proposed BioMiCo, a two-level hierarchical Bayes model of a mixture of multidimensional distributions constrained by Dirichlet priors to identify each OTU cluster, called an assemblage, and to estimate the mixing ratio of the assemblages within a sample. Another popular method for learning community structure is non-negative matrix factorization (NMF) [14, 15]. Cai et al. [16] proposed a supervised version of NMF to identify communities representing the connection between the sample microbial composition and OTUs and to infer systematic differences between different types of communities.

Knights et al. [12] reviewed how these statistical methods can be applied to microbial data. However, the methods for identifying the relationship between bacterial communities and environmental factors are not well developed.

These methods are useful in a variety of circumstances, but they also possess limitations. Both BioMiCo and supervised NMF can associate only one categorical variable to the microbial community. To our knowledge, no framework currently exists that adequately details the interaction between a mixture of microbial communities and multiple environmental factors. A new framework is needed to address this problem.

To remedy this situation, we propose a novel approach, called BALSAMICO (BAyesian Latent Semantic Analysis of MIcrobial COmmunities). The contributions of our research are as follows:

- BALSAMICO uses the OTU abundances and the host environmental factors as input to provide a path to interpret microbial communities and their environmental factors. In BALSAMICO, the data matrix of a microbiome is approximated by the product of two matrices. One matrix represents a mixing ratio of microbial communities, and the other matrix represents the abundance of bacteria in each community. BALSAMICO decomposes the mixing ratio into the observed environmental factors and their coefficients in order to identify the influence of the environmental factors.
- Not only is this decomposition a part of ordinary NMF, but it improves upon ordinary NMF by displaying a hierarchical structure. One clear advantage of the Bayesian hierarchical model is to introduce stochastic fluctuations at all levels. This makes it possible to smoothly handle missing data and to easily give credible intervals.
- BALSAMICO does not require prior knowledge regarding the communities to which the bacteria belong. BALSAMICO can estimate an unknown community structure without explicitly using predetermined community information. Furthermore, the parameters of unknown community structures can be estimated automatically through Bayesian learning.
- While the computation cost of other methods, which use Gibbs sampling, is high, we provide an efficient learning procedure for BALSAMICO by using a variational Bayesian inference and Laplace approximation to reduce computational cost. The software package that implements BALSAMICO in the R environment is available from GitHub (https://github.com/abikoushi/BALSAMICO).

The structure of this paper will proceed as follows: The “Methods” section describes our model and the procedure for parameter estimation. The “Results” section contains an evaluation of the accuracy of the estimator using synthetic data. Additionally, BALSAMICO is applied to clinical metagenomic data to detect bacterial communities related to colorectal cancer (CRC). Through this content, both the usefulness and accuracy of BALSAMICO are confirmed.

## Implementation

Calculations for this method are based on the assumption that the microbiome consists of several communities. BALSAMICO extracts the communities from the data, using NMF. Suppose that we observe a non-negative integer matrix ***Y***=(*y*_*n*,*k*_) (*n*=1,…,*N*,*k*=1,…,*K*), where *y*_*n*,*k*_ is the microbial abundance of *k*-th taxon in the *n*-th sample. Our goal is to seek a positive *N*×*L* matrix ***W*** and an *L*×*K* matrix ***H***, such that

1 $$\begin{array}{*{20}l} \boldsymbol{Y} \approx \boldsymbol{W}\boldsymbol{H}.  \end{array} $$

The (*n*,*l*)-element *w*_*n*,*l*_ of matrix ***W*** can be interpreted as contributing to community *l* of sample *n*. The (*l*,*k*)-element *h*_*l*,*k*_ of matrix ***H*** can be interpreted as the relative abundance of the *k*-th taxon given community *l*. We thus refer to ***W*** as the *contribution matrix* and to ***H*** as the *excitation matrix*.

**Fig. 1:**
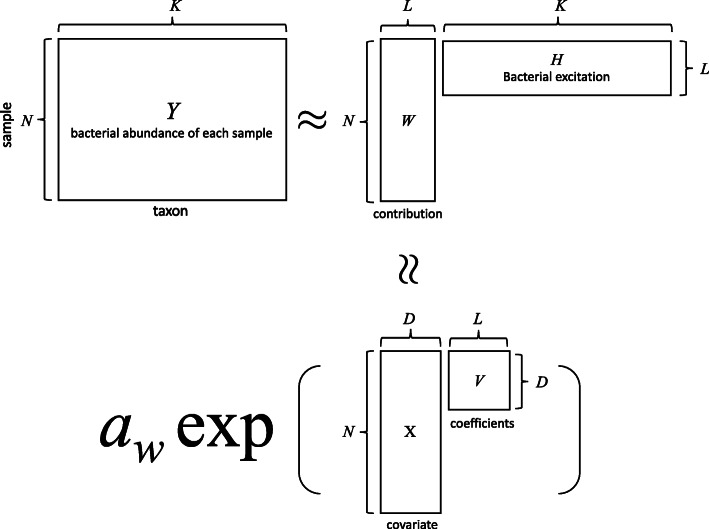
Conceptual diagram of matrix factorization in BALSAMICO

In addition, if covariate ***X***=(*x*_*n*,*d*_) (*d*=1,…,*D*) is observed (e.g. whether or not the *n*-th sample has a certain disease), our aim is to investigate how ***W*** changes when ***X*** is given. For this, BALSAMICO seeks the *D*×*L* matrix ***V***, such that

2 $$\begin{array}{*{20}l} \boldsymbol{W} \approx a_{w} \exp(\boldsymbol{X}\boldsymbol{V})  \end{array} $$

where *a*_*w*_ is a shape parameter of gamma distribution and exp(·) is an element-wise exponential function. As shown in Fig. 1, BALSAMICO approximates matrix ***Y*** using the product of low-rank matrices.

In brief, we consider the following hierarchical model:

3 $$\begin{array}{*{20}l} \boldsymbol{h}_{l} & \sim \text{Dirichlet}(\boldsymbol{\alpha}), \end{array} $$

4 $$\begin{array}{*{20}l} \boldsymbol{B} &= \exp(-\boldsymbol{X} \boldsymbol{V}) \end{array} $$

5 $$\begin{array}{*{20}l} w_{n,l} & \sim \text{Gamma}(a_{w}, B_{n,l}), \end{array} $$

6 $$\begin{array}{*{20}l} t_{n,l} &\sim \text{Poisson}(w_{n,l} \tau_{n}),  \end{array} $$

7 $$\begin{array}{*{20}l} \boldsymbol{s}_{n,l} &\sim \text{Multinomial}(t_{n,l}, \boldsymbol{h}_{l}) \end{array} $$

8 $$\begin{array}{*{20}l} y_{n,k} &= \sum_{l=1}^{L}s_{n,l,k}.  \end{array} $$

*B*_*n*,*l*_ is the (*n*,*l*)-element of matrix ***B***,*s*_*n*,*l*,*k*_ is the *k*-th element of vector ***s***_*n*,*l*_,*τ*_*n*_ is an offset term, ***V*** is a *D*×*L* matrix, and ***S***={*s*_*n*,*l*,*k*_} are latent variables. The variable ***S*** is introduced for inference to make the calculations more smooth. In this study, we set $\tau _{n}=\sum _{k=1}^{K}y_{n,k}$. The total read count *τ*_*n*_ is dependent on the setting of the DNA sequencer, so it is not a reflection of an abundance of bacteria. The offset term then adjusts the setting-based effect on the read counts to accurately estimate ***W***. The (*d*,*l*)-element *v*_*d*,*l*_ of matrix ***V*** can be interpreted as contributing to the community *l* of the *d*-th covariate. This Poisson observation model is frequently used in Bayesian NMF [17]. The Gamma distribution is a conjugate prior for the Poisson distribution and the Dirichlet distribution is the conjugate prior for the multinomial distribution.

**Fig. 2:**
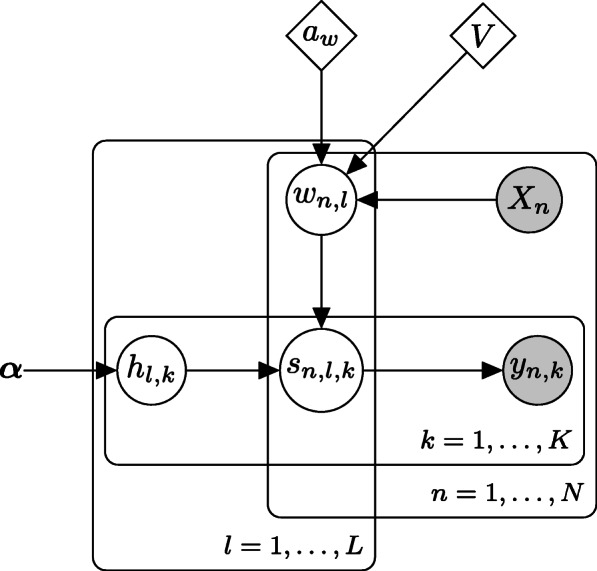
Plate diagram of the data generating process in BALSAMICO. The white nodes indicate latent variables and the gray nodes indicate observed variables. The parameters represented by diamonds are estimated by Laplace approximation

Figure 2 shows a plate diagram of the data generating process. BALSAMICO estimates parameters ***W***,***H***,*a*_*w*_, and ***V***, using variational inference [18]. More details for this parameter estimation procedure are listed in the supplemental document. After estimating the parameters it is possible to move on to analyzing real data, but first the accuracy of the estimation should be confirmed.

## Results

### Simulation study using gamma distribution

Starting with the BALSAMICO estimated parameters detailed in “Methods,” we can now evaluate these parameters for accuracy before moving on to an analysis of real-world data. The following simulation experiments evaluate the bias, the standard deviation (SD), and the coverage probability (CP) of the estimators. The bias of $\hat \theta $ is defined by the difference between the true value and the estimated value $(E[\hat {\theta }]-\theta)$. The coverage probability is the proportion at which the 95% credible interval contains the true value. The synthetic data was naturally produced via the data generating process given by Eqs. 3–8.

We estimated the parameters in 10,000 replicates of the experiment. We set *X*=(***1***,***x***_1_,***x***_2_), where ***1*** is a vector of ones. The variables ***x***_1_ and ***x***_2_ are sampled independently from a standard normal distribution and a Bernoulli distribution with a probability of 0.5, respectively. When generating the synthetic data, we set *N*=100,*K*=100,*L*=3,*τ*_*n*_=10,000, and *α*_*k*_=1 for all *k*. We also set *α*_*k*_=1 for all *k* when estimating parameters, which is equivalent to a non-informative prior distribution. To avoid the problem of label switching [19], the estimated parameters are rearranged as *v*_21_≤*v*_22_≤*v*_23_.

The gamma distribution changes considerably when the shape parameter *a*_*W*_ is smaller than 1, which leads to a heavier tail than an exponential distribution. Consequently, we conducted two patterns of the simulation. Table 1 shows these results. The first half of the table shows the case of a heavy tail.

**Table 1:** Bias, SD, and CP of the estimates

	True value	Bias	SD	CP
*a*_*w*_	0.5	-0.01	0.10	
*v*_11_	**1.00**	**0.00**	**0.30**	**0.86**
*v*_12_	-0.50	-0.00	0.15	0.95
*v*_13_	0.50	0.00	0.30	0.94
*v*_21_	**1.00**	**0.01**	**0.30**	**0.86**
*v*_22_	0.00	0.00	0.15	0.95
*v*_23_	0.00	0.00	0.30	0.94
*v*_31_	**1.00**	**0.01**	**0.30**	**0.86**
*v*_32_	0.50	0.00	0.15	0.95
*v*_33_	0.50	0.01	0.29	0.95
*a*_*w*_	2.00	0.06	0.17	
*v*_11_	**1.00**	**-0.04**	**0.13**	**0.93**
*v*_12_	-0.50	-0.00	0.07	0.94
*v*_13_	0.50	0.00	0.15	0.94
*v*_21_	**1.00**	**-0.04**	**0.13**	**0.92**
*v*_22_	0.00	0.00	0.07	0.94
*v*_23_	0.00	0.00	0.15	0.94
*v*_31_	**1.00**	**-0.03**	**0.13**	**0.94**
*v*_32_	0.50	-0.00	0.07	0.94
*v*_33_	-0.50	0.01	0.15	0.95

The parameters in boldface is the intercepts

When the shape parameter *a*_*w*_ is set to 0.5, the credible intervals of *v*_*i*1_ (*i*=1,2,3) have under-coverage. However, this was only observed in intercept terms. In most cases, the CP was almost equal to the nominal value. This result indicates that there is no inconsistency when interpreting the estimated coefficients.

Moreover, the parameters were estimated with small biases. By this we know that the proposed method produces reasonable estimates.

### Simulation study for model selection

**Fig. 3:**
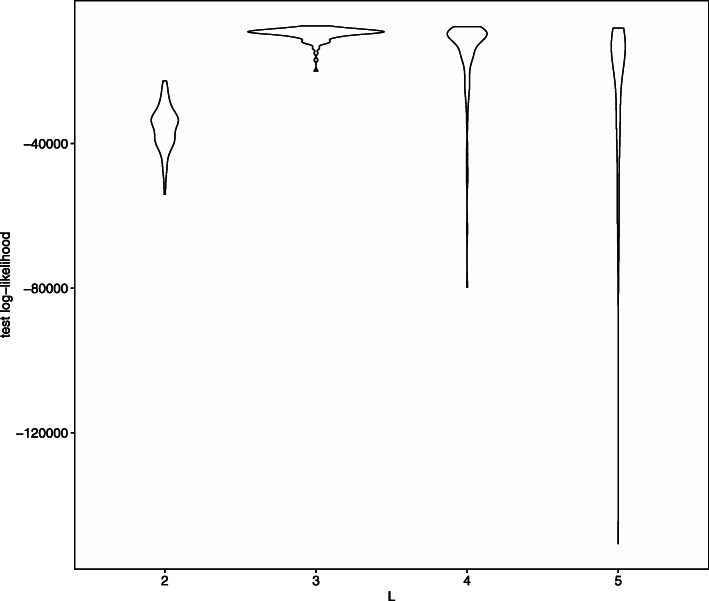
Mean of test log-likelihood evaluated by 10-fold cross-validation. The *x*-axis corresponds to the number of communities *L*

Next, we evaluate model selection by cross-validation. When generating the synthetic data, we set *L*=3 and *a*_*W*_=1. Other settings were the same as the previous sub-section. We select the number of communities by the 10-fold cross validation in each trial. In all 100 trials, *L*=3 was selected for all 100 times. Figure 3 shows the distribution of the mean of the test log-likelihood n each trial.

### Simulation study under a more complicated situation

To investigate the behavior of the estimates in more complex cases, we also conducted a simulation with a larger number of explanatory variables and communities. We estimated the parameters in 100 replicates of the experiment. We set *X*=(***1***,***x***_1_,***x***_2_,***x***_3_), where ***1*** is a vector of ones. The variables ***x***_1_ are sampled from standard normal distribution. The variables ***x***_2_ and ***x***_3_ are independent and follow a Bernoulli distribution with a probability of 0.5. When generating the synthetic data, we set *L*=7. The coefficients *v*_*d*,*l*_ were generated independently following from a standard normal distribution. Other settings were the same as the previous sub-section.

**Fig. 4:**
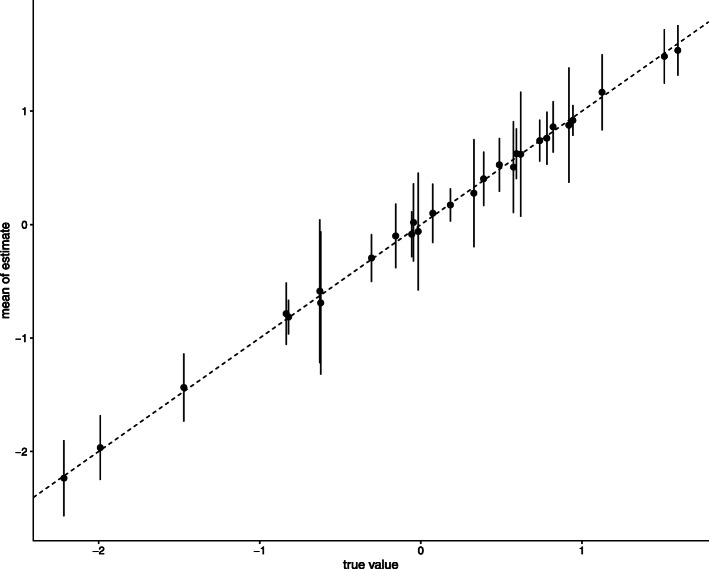
The comparison true ***V*** and the mean of estimates $\boldsymbol {\hat V}$. The error bars indicate standard deviation

Figure 4 shows the comparison between the estimates and the true value of ***V***. We found that the mean of the estimates is close to the true values. The coverage probabilities are shown in the Supplemental Table S1.

### Simulation study using other distributions

We conducted two simulations to assess the sensitivity of BALSAMICO. We generated ***W*** from a distribution other than the gamma distribution and evaluated the behavior of the estimates of ***V***. Since ***W*** is a non-negative matrix, we use lognormal and Weibull distribution. In the lognormal case, we set the log-mean parameters to ***XV*** and the log-variance parameter to 1. In the Weibull case, we set the shape parameter to 2, and the scale parameters to exp(***XV***). Other settings were the same as the sub-section “Simulation study using gamma distribution”. We estimated the parameters in 100 replicates of the experiment. Tables 2-3 show these results. It can be seen that the estimated values of the intercept terms have a large bias, but the estimated values of the coefficients are close to true values. This result indicates that our approach is robust to the misspecification of the underlying model.

**Table 2:** mean and SD of the estimates (using lognormal distribution)

	True	Mean	SD
*a*_*w*_		1.18	0.11
*v*_11_	**1.00**	**1.27**	**0.22**
*v*_12_	-0.50	-0.50	0.12
*v*_13_	0.50	0.55	0.28
*v*_21_	**1.00**	**1.28**	**0.24**
*v*_22_	0.00	-0.03	0.12
*v*_23_	0.00	-0.01	0.27
*v*_31_	**1.00**	**1.33**	**0.22**
*v*_32_	0.50	0.48	0.13
*v*_33_	-0.50	-0.49	0.27

The parameters in boldface is the intercepts

**Table 3:** mean and SD of the estimates (using Weibull distribution)

	True	Mean	SD
*a*_*w*_		3.28	0.28
*v*_11_	**1.00**	**-0.31**	**0.10**
*v*_12_	-0.50	-0.51	0.06
*v*_13_	0.50	0.50	0.11
*v*_21_	**1.00**	**-0.31**	**0.12**
*v*_22_	0.00	0.00	0.05
*v*_23_	0.00	-0.01	0.11
*v*_31_	**1.00**	**-0.31**	**0.11**
*v*_32_	0.50	0.49	0.06
*v*_33_	-0.50	-0.49	0.11

The parameters in boldface is the intercepts

This being confirmed, it is now possible to apply the proposed method to real data to assess how well it conforms to current studies.

### Results on real data

#### Zeller’s data

This section tests the usefulness of our results by investigating the identification of gut dysbiosis associated with the development of CRC. Zeller et al. [20] studied gut metagenomes extracted from 199 persons: 91 CRC patients, 42 adenoma patients, and 66 controls. The data is available in the R package “curatedMetagenomicData” (https://github.com/waldronlab/curatedMetagenomicData). This analysis uses the abundance of genus-level taxa.

**Fig. 5:**
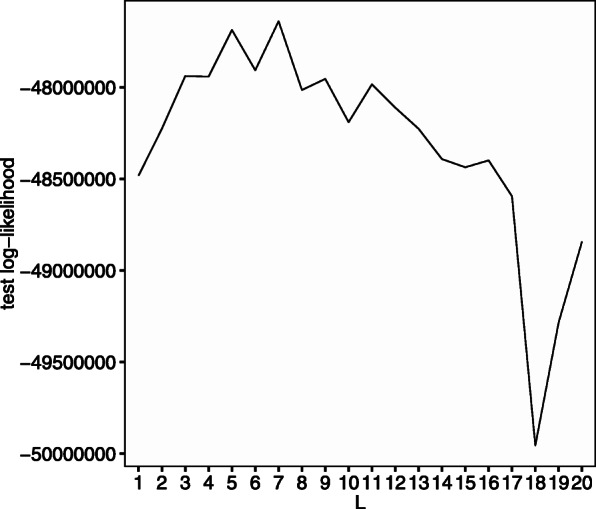
Mean of test log-likelihood evaluated by leave-one-out cross-validation (Zeller’s data). The *x*-axis corresponds to the number of communities *L*

We set *α*_*k*_=1 and use the disease label, gender, and age as covariates. The age variable is scaled by dividing by 100. The number of communities *L*=7 was selected using leave-one-out cross-validation (Fig. 5).

**Fig. 6:**
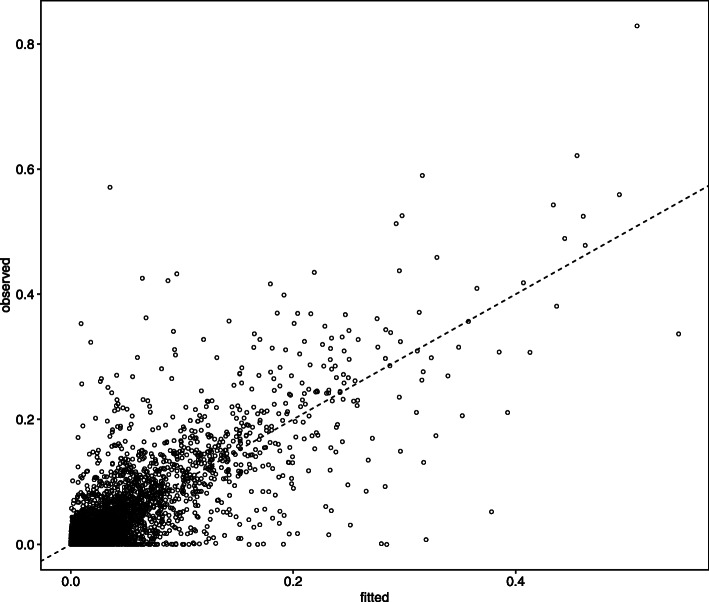
Comparison between ***WH*** (fitted) and normalized abundance (observed)

Figure 6 shows the estimated ***WH*** and normalized abundance $(y_{n,k}/\{\sum _{k=1}^{L} y_{n,k}\})$. The observed data matrix is approximated by ***WH***.

**Fig. 7:**
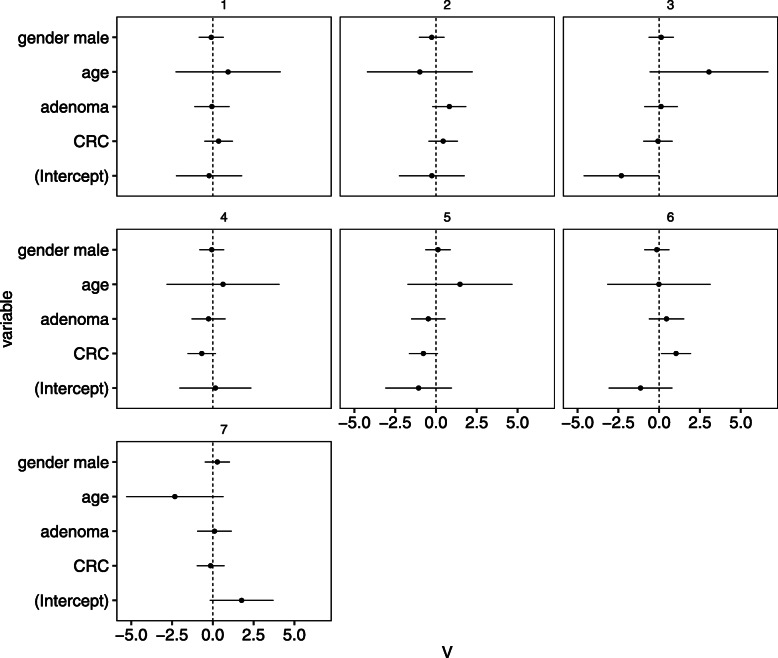
Estimated coefficients ***V*** for the environmental factors (Zeller’s data). The each panel corresponds to community, the *x*-axis corresponds to the value of coefficients and the *y*-axis corresponds to the variable name. Where the term of “intercept” means constants not depends on explanatory variables. The bars indicate 95%-credible intervals

Figure 7 shows estimates of coefficient ***V***. First, we can see that the human microbiome is not significantly dependent on gender as the absolute value of coefficients for gender is small, and their credible intervals contain zero. It can be seen that the coefficient of the variable “age” has a large confidence interval. We examined the results of removing the variable “age” and found that the coefficients for the other variables did not change significantly (Supplementary Figure S1). Focusing on CRC, we can see that the credible intervals of the coefficient for community 6 do not contain zeros. Moreover the value of coefficients for community 6 increases as adenoma progresses to CRC. Community 6 is thus strongly suspected of being associated with the disease.

**Fig. 8:**
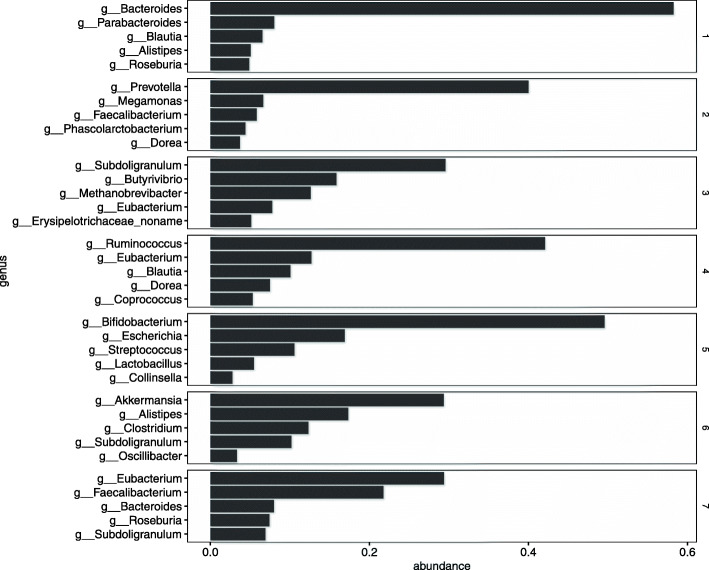
Estimated excitation matrix ***H*** (Zeller’s data). Five most frequently occurring genus in each community

Figure 8 shows the top five estimates of *h*_*l*,*k*_ in each community *l*. Arumugam et al. [21] reports that the human gut microbiome can be classified into several types, called enterotypes. Arumugam et al. [21] shows that an enterotype is characterized by the differences in the abundance of *Bacteroides*, *Prevotella*, and *Ruminococcus*. Communities 1, 2, and 4 are characterized by an abundance of *Bacteroides*, *Prevotella*, and *Ruminococcus* respectively (Fig. 8). Communities 1, 2, and 4 may be enterotype-like clusters.

Community 6, which is suspected of being associated with CRC, is characterized by abundant *Akkermansia*. This is markedly different from the other communities and deserves further examination. We examined the results of changing the number of communities *L* to 6 or 8, and found that major genus of Community 6, which is suspected of being related to CRC is not significantly changed (Supplementary Figures S2–S4))

To detect the bacteria that exist exclusively in community 6, we use the following quantity:

9 $$\begin{array}{*{20}l} \eta_{l,k} = \frac{h_{l,k}}{\sum_{l=1}h_{l,k}}.  \end{array} $$

*η*_*l*,*k*_ is the ratio of the relative abundance of bacteria *k* in community *l* to that of other communities.

The bacteria belonging to community 6 are suspected of being associated with CRC. Table 4 shows estimates of *η*_6,*k*_ greater than 0.95. This result indicates that these bacteria are related to CRC. These bacteria that characterize community 6 are *Akkermansia*, *Desulfotomaculum*, *Mucispirillum*, *Methanobacterium*, *Hahellaceae*, *Nakaseomyces*, *Fretibacterium*, *Alphabaculovirus*, *Synergistes*, and *Enhydrobacte*. The connection between these bacteria and CRC is further supported by current studies.

- *Akkermansia*: Weir et al. [22] reports that mucin-degrading bacteria, *Akkermansia muciniphila*, was present in a significantly greater proportion in the feces of colon cancer patients. This is consistent with our result.
- *Desulfotomaculum*: *Desulfotomaculum* belongs to sulfate-reducing bacteria, which obtains energy by oxidizing organic compounds or molecular hydrogen while reducing sulfate to hydrogen sulfide. Hydrogen sulfide is toxic to intestinal epithelium cells and causes DNA damage in human cells [23].
- *Mucispirillum*: Similar to *Akkermansia*, *Mucispirillum* is a mucus-resident bacteria and may coexist with *Akkermansia*. If so, these bacteria are distributed in the mucus layer that covers the mucous membrane of the intestine [24].
- *Methanobacterium*: Patients with CRC contain a higher proportion of breath methane excreters than the control group [25]. *Methanobacterium* is a methanogenic bacterium.
- *Enhydrobacter*: Xu & Jiang [26] apply linear discriminative analysis to biomarker discovery. The result suggests that *Enhydrobacter* can be a biomarker for CRC.

**Table 4:** Estimates of *η*_6,*k*_ greater than 0.95

Genus	*η*
Synergistes	1.000
Methanobacterium	1.000
Desulfotomaculum	1.000
Nakaseomyces	1.000
Fretibacterium	1.000
Akkermansia	1.000
Alphabaculovirus	0.999
Enhydrobacter	0.999
Mucispirillum	0.998
Hahellaceae_unclassified	0.998

The information found in the above studies strongly supports the results returned by applying our method to real data. This suggests that BALSAMICO is able to successfully and accurately analyze communities of bacteria and their environmental interactions.

Finally, we compare results for *L*=5,*L*=6 and *L*=7. Supplemental Figures S2 and S3 show the the results for *L*=5. Figure S4 and S5 show the the results for *L*=5. When *L*=5, the community 1 is positively correlated with CRC. The major genera of community 1 include *Akkermansia* and *Alistipes*. This trend is consistent with the result for *L*=7. When *L*=6, the major genera of community 6 include *Akkermansia* and *Alistipes* and the community 6 is positively correlated with CRC.

#### David’s data

David et al. [27] studied longitudinal fecal metagenome from two Donors A and B. Donor A went on a trip abroad in days 71 to 122 and donor B has enteric infection in days 151 to 159. The data is available in the R package “themetagenomics”. We analyze David’s dataset using BALSAMICO to investigate the bacteria associated with food poisoning and the changes in microbiome after food poisoning. In this analysis, we regard donor A as a baseline. This analysis uses the abundance of genus-level taxa. We set *α*_*k*_=1 and use the donor label, date, and the interaction of these as covariates. The date variable is coded as intervals (0,50], (50, 100], (150,200], and (200, 364]. Although BALSAMICO could treat date as a continuous predictor, the effect of time is likely to be non-linear, so we prefer to treat time as a categorical variable. Results for an analysis where we treat time as a continuous variable are in Supplemental Figures S6–S8.

**Fig. 9:**
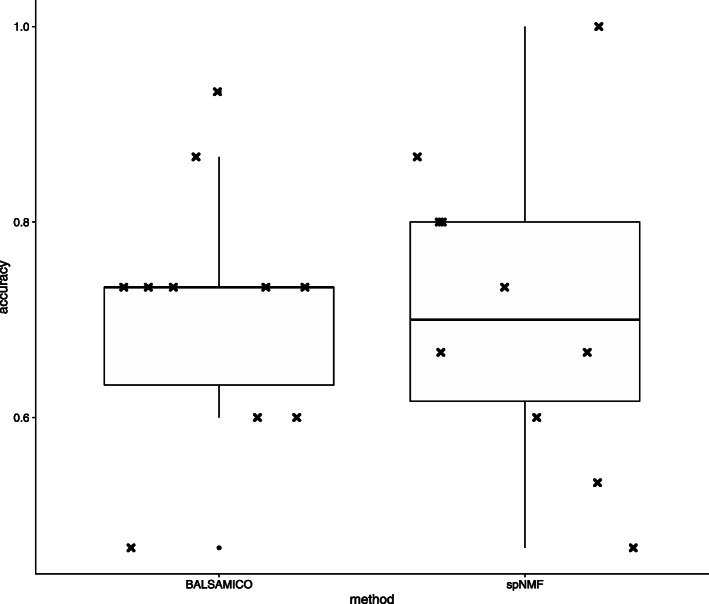
Mean of test log-likelihood evaluated by leave-one-out cross-validation (David’s data). The *x*-axis corresponds to the number of communities *L*

**Fig. 10:**
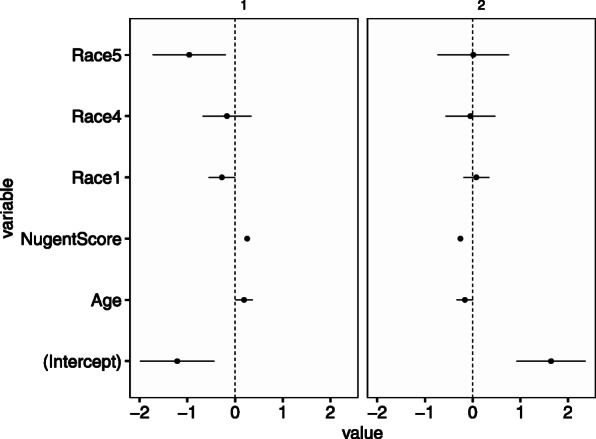
Estimated coefficients ***V*** for the environmental factors (David’s data). The each panel corresponds to community, the *x*-axis corresponds to the value of coefficients and the *y*-axis corresponds to the variable name. Where the term of “intercept” means constants not depends on explanatory variables. The bars indicate 95%-credible intervals

The number of communities *L*=6 was selected using 10-fold cross-validation (Fig. 9). Figure 10 shows estimates of coefficient ***V***. We observed that, in the period of (150,200] corresponding to the period when donor B suffered food poisoning, the abundance of community 6 in Donor B increased (the coefficient for “DonorB:T(150,200]” at the community 6 is large and its credible interval does not contain zero). Furthermore, Donor A was exposed to a novel diet and environment while traveling and had diarrhea on days 80 to 85 and 104 to 113. Corresponding to this fact, the coefficient of the baseline of community 6 is large in the periods (50, 100] and (100, 150].

**Fig. 11:**
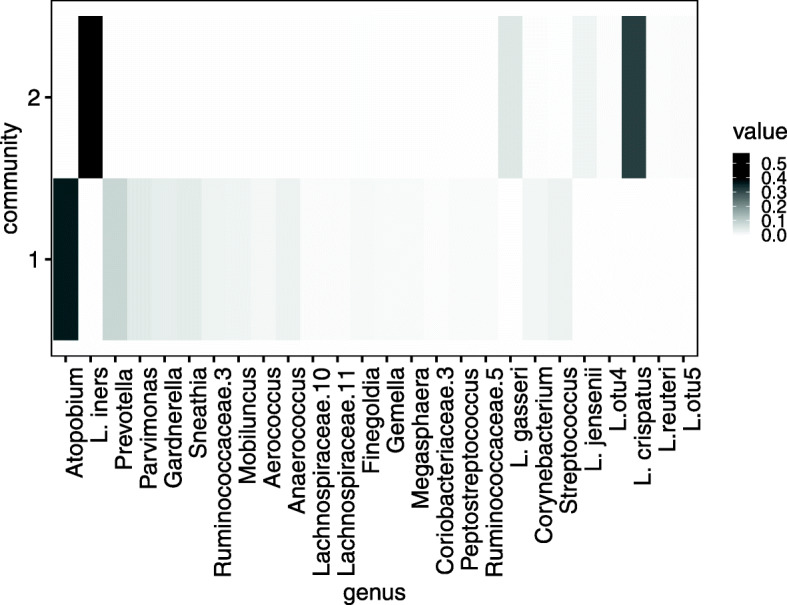
Estimated excitation matrix ***H*** (David’s data). Five most frequently occurring genus in each community

Figure 11 shows the top five estimates of *h*_*l*,*k*_ in each community *l*. Community 6 is characterized by abundant *EscherichiaShigella* and *Salmonella*. These bacteria cause food poisoning. David et al. reported that donor B had a Salmonella infection and reads from the *Enterobacteriaceae* (which include *EscherichiaShigella*) increased during donor B’s infection [27]. Our result is consistent with this diagnosis.

Next, the abundance of community 3 at donor B increases in (150,200] and (200, 364] corresponding to the period after the treatment of food poisoning. Community 3 is characterized by abundant *Lachnospiraceae*. The results show that food poisoning and its treatment changed the composition of the microbiome.

### Comparison with other state-of-the-art methods

#### Comparison with bioMiCo

We also compared the results of BALSAMICO with those of BioMiCo [13] using Gajer’s data [28] which they analyzed. This dataset consists of vaginal microbiome samples from 32 women at different time points (a total of 889 samples), together with the Nugent score [29], which is a measure of bacterial vaginosis for each sample. We used this Nugent score, age, and race (Black=0, White=1, Hispanic=5, and others=4). The age variable was scaled by dividing by 10. We set *α*_*k*_=1. To simplify comparison with those of BioMiCo, we set the number of communities to 2.

**Fig. 12:**
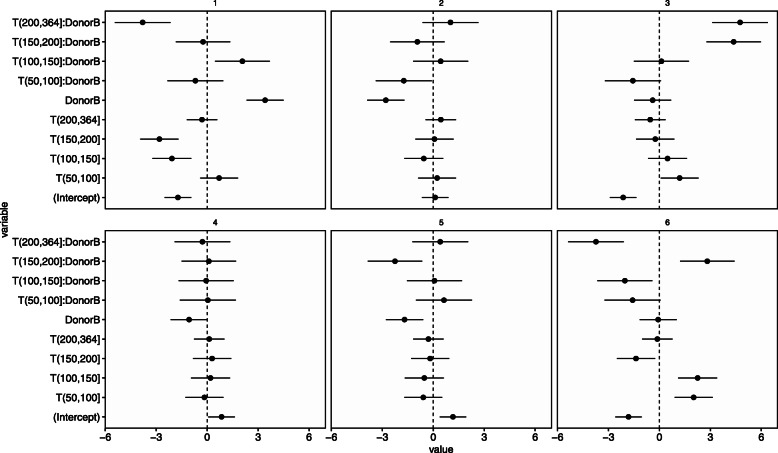
Estimated coefficients ***V*** for the environmental factors (Gajer’s data). The each panel corresponds to community, the *x*-axis corresponds to the value of coefficients and the *y*-axis corresponds to the variable name. Where the term of “intercept” means constants not depends on explanatory variables. The bars indicate 95%-credible intervals

**Fig. 13:**
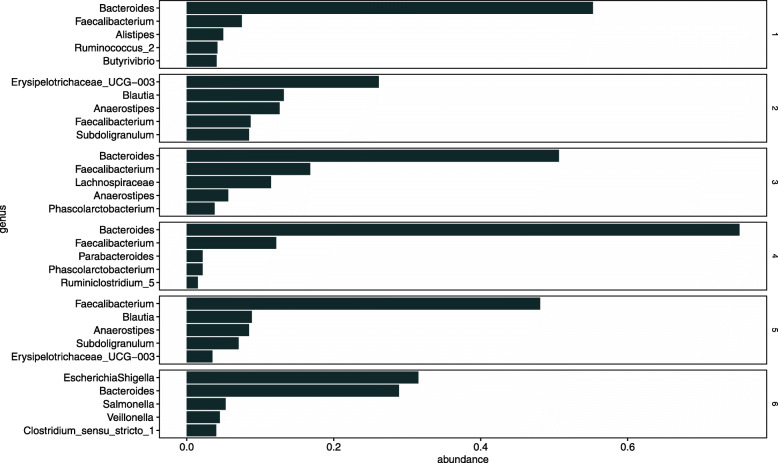
Estimated excitation matrix ***H*** (Gajer’s data). The *x*-axis corresponds to the community, and the *y*-axis corresponds to the genus. The black parts indicate high abundance, and the white parts indicate zero

Figures 12 and 13 show the estimates of ***V*** and ***H***, respectively. Figure 12 shows the samples with the categories “intermediate” and “high” have a high proportion of community 1 and a low proportion of community 2. Although the result of BALSAMICO is very close to that of BioMiCo, BALSAMICO provides more useful sample-level information compared with BioMiCo. For example, BALSAMICO shows that the samples with the race “Hispanic” have a low proportion of community 1 and community varies greatly by sample age.

#### Comparison with supervised NMF

We evaluated the performance of BALSAMICO with other state-of-the-art methods on the real data.

We first compared the results of BALSAMICO with those of the supervised NMF [16] using Zeller’s data. We used the R package SpNMF with default settings. Since SpNMF can only handle binary responses, we exclude adenoma samples and coded 0 for healthy controls and 1 for CRC patients. The number of communities was selected by Cai’s proposed method which is implemented as an R function “chty”.

As a result, SpNMF detected two microbial communities related to CRC. Table 5 shows the five most abundant genera in each community and each response. As can be seen from the table, community 1 for CRC is quite similar to community 1 for control. SpNMF is not a method of calculating the importance or significance of variables. Thus, in order to interpret this results, we should perform another analysis such a logistic regression using the obtained feature quantities ***W***.

**Table 5:** Five most frequently occurring genus in each community and each response (Zeller’s data)

Label	CRC		Control	
Community	1	2	1	2
	Bacteroides	Akkermansia	Bacteroides	Ruminococcus
	Eubacterium	Prevotella	Eubacterium	Bifidobacterium
	Subdoligranulum	Escherichia	Faecalibacterium	Streptococcus
	Ruminococcus	Methanobrevibacter	Ruminococcus	Eubacterium
	Faecalibacterium	Butyrivibrio	Subdoligranulum	Blautia

**Fig. 14:**
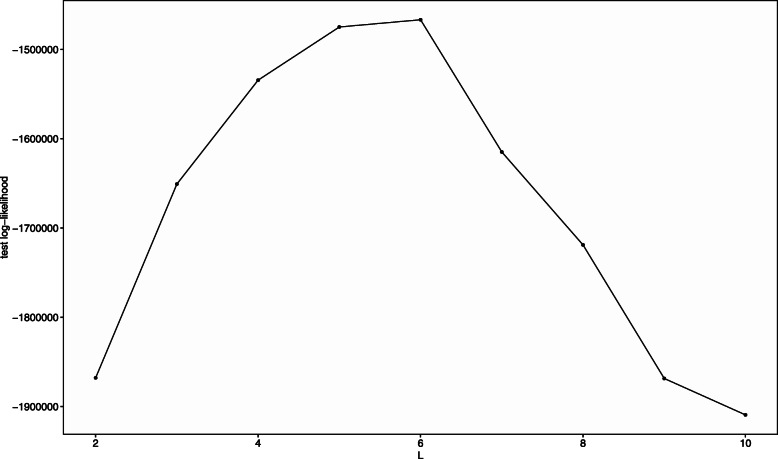
Accuracy with 10-fold cross validation by logistic regression (Zeller’s data)

To compare the goodness of feature extraction, logistic regression was performed using the contribution matrix ***W*** obtained by BALSAMICO and SpNMF as the explanatory variable. However, because the matrix *W* from BALSAMICO is constrained by $\sum _{l=1}^{L} w_{n,l} \approx 1$ for all *n*, *w*_*n*,7_ is removed as an explanatory variable. We classified CRC or healthy and evaluate the accuracy with 10-fold random cross validation. The results are shown in Fig. 14. The mean accuracy was 0.71 for both methods.

Tables 6–7 show the regression coefficients of logistic regression and these *p*-values of Wald test. From the Table 7, community 2 for control in the Table 5 are negatively correlated with CRC. The community 2 for “CRC” in SpNMF is a little similar to community 6 in BALSAMICO. However the *p*-value of this variable in logistic regression is not significant at the 5% level. On the other hand, from the Table 6, community 6 in BALSAMICO is positively correlated with CRC. This result is consistent with previous sub section.

**Table 6:** Regression coefficients by logistic regression using BALSAMICO (Zeller’s data)

Variable	Estimate	*P*-value
(Intercept)	0.023	0.972
*W*_*n*,1_	2.002	0.082
*W*_*n*,2_	1.180	0.384
*W*_*n*,3_	1.057	0.357
*W*_*n*,4_	-2.493	0.053
*W*_*n*,5_	-3.234	0.048
*W*_*n*,6_	5.144	0.007

**Table 7:** Regression coefficients by logistic regression using SpNMF (Zeller’s data)

Variable	Estimate	*P*-value
(Intercept)	0.246	0.572
*W*_*n*,1_ (CRC 1)	1.24×10^−8^	0.051
*W*_*n*,2_ (CRC 2)	3.37×10^−8^	0.061
*W*_*n*,3_ (control 1)	− 6.72×10^−9^	0.235
*W*_*n*,3_ (control 2)	− 3.30×10^−8^	0.011

Next, we compare between supervised NMF and BALSAMICO on David’s data. In the David’s data, we conducted two analyses. First, we coded donor A and B as 0 and 1, respectively. Next, we exclude donor A and coded 0 for pre-infection term (days 0 to 150) and 1 for post-infection term (days 151 to 364). As a result of first analysis, SpNMF detected four microbial communities which characterized donor A and two communities which characterized donor B. Table 8 shows the five most abundant genera in each community and each response.

**Table 8:** Five most frequently occurring genus in each community and each response (first analysis of David’s data)

label	Donor B	
community	1	2
	Bacteroides	Bacteroides
	Faecalibacterium	Faecalibacterium
	Lachnospiraceae	Alistipes
	Anaerostipes	Butyrivibrio
	Phascolarctobacterium	Coprococcus_2
label	Donor A	
community	1	2
	Faecalibacterium	EscherichiaShigella
	Anaerostipes	Bacteroides
	Blautia	Salmonella
	Subdoligranulum	Clostridium_sensu_stricto_1
	Erysipelotrichaceae_UCG-003	Veillonella
label	Donor A	
community	3	4
	Bacteroides	Erysipelotrichaceae_UCG-003
	Faecalibacterium	Blautia
	Parabacteroides	Anaerostipes
	Phascolarctobacterium	Faecalibacterium
	Ruminiclostridium_5	Subdoligranulum

As the same manner above, we perform logistic regression using the contribution matrix ***W*** obtained by BALSAMICO and SpNMF as the explanatory variable. We classified donor A or B and evaluate the accuracy with 10-fold random cross validation. The mean accuracy was 0.96 for both methods.

Tables 9–10 show the regression coefficients of logistic regression and these *p*-values of Wald test.

**Table 9:** Regression coefficients by logistic regression using SpNMF (first analysis of David’s data)

Variable	Estimate	*P*-value
(Intercept)	-0.902	0.016
*W*_*n*,1_ (donor B)	1.14×10^−4^	0.000
*W*_*n*,2_ (donor B)	1.60×10^−4^	0.000
*W*_*n*,3_ (donor A)	− 5.70×10^−5^	0.010
*W*_*n*,4_ (donor A)	− 1.51×10^−5^	0.488
*W*_*n*,5_ (donor A)	− 8.04×10^−6^	0.361
*W*_*n*,6_ (donor A)	− 4.14×10^−4^	0.024

**Table 10:** Regression coefficients by logistic regression using BALSAMICO (first analysis of David’s data)

Variable	Estimate	*P*-value
(Intercept)	5.690	0.000
*W*_*n*,2_	-23.724	0.000
*W*_*n*,3_	0.925	0.652
*W*_*n*,4_	-7.061	0.000
*W*_*n*,5_	-10.113	0.000
*W*_*n*,6_	-6.457	0.000

In the second analysis, SpNMF detected two microbial communities related to post-infection term. Table 11 shows the five most abundant genera in each community and each response. We perform logistic regression using the contribution matrix ***W*** obtained by BALSAMICO and SpNMF as the explanatory variable. We classified pre or post infection and evaluate the accuracy with 10-fold random cross validation. The mean accuracy was 0.98 for both methods.

**Table 11:** Five most frequently occurring genus in each community and each response (second analysis of David’s data)

Label	Post		Pre	
Community	1	2	1	2
	EscherichiaShigella	Bacteroides	Bacteroides	Bacteroides
	Bacteroides	Faecalibacterium	Butyrivibrio	Faecalibacterium
	Anaerostipes	Lachnospiraceae	Alistipes	Coprococcus_2
	Blautia	Anaerostipes	Faecalibacterium	Alistipes
	Salmonella	Phascolarctobacterium	Ruminococcus_2	Ruminococcus_2

Tables 12–13 show the regression coefficients of logistic regression and these *p*-values of Wald test. The community 1 for “post” in SpNMF is a little similar to community 6 in BALSAMICO. However the *p*-value of this variable in logistic regression is not significant at the 5% level. Thus, if we have no prior knowledge, we may not noticed that this community associated with food-poisoning.

**Table 12:** Regression coefficients by logistic regression using SpNMF (second analysis of David’s data)

Variable	Estimate	*P*-value
(Intercept)	-2.388	0.009
*W*_*n*,1_ (post)	8.29×10^−4^	0.635
*W*_*n*,2_ (post)	2.04×10^−4^	0.003
*W*_*n*,3_ (pre)	− 4.05×10^−5^	0.102
*W*_*n*,4_ (pre)	2.85×10^−5^	0.066

**Table 13:** Regression coefficients by logistic regression using BALSAMICO (second analysis of David’s data)

Variable	Estimate	*P*-value
(Intercept)	-9.842	0.002
*W*_*n*,2_	51.729	0.038
*W*_*n*,3_	18.012	0.006
*W*_*n*,4_	12.686	0.005
*W*_*n*,5_	20.053	0.132
*W*_*n*,6_	8.936	0.007

Finally, linear regression was performed using the contribution matrix ***W*** obtained by BALSAMICO and SpNMF as the explanatory variable. We predict sample days and evaluate the root mean squared error (RMSE) with 20-fold random cross validation. As above, in applying SpNMF, we set the pre-infection term as 0, and the post-infection term as 1. The means and standard deviations of the RMSE were 43.2 and 11.2 for BALSAMICO and 48.3 and 10.7 for SpNMF, respectively. To compare the RMSE of the two methods, we performed paired *t*-test and paired Wilcoxon test and the *p*-values were 0.026 and 0.044, respectively.

To confirm the affect of the sequencing depth, we have sampled *y*_*n*,*k*_ from the empirical distribution, set total read count *τ*_*n*_ to 10000, and performed the same regression on David’s data. The means and standard deviations of the RMSE were 42.7 and 8.1 for BALSAMICO and 48.3 and 10.7 for SpNMF, respectively. To compare the RMSE of the two methods, we performed paired t-test and paired Wilcoxon test and the *p*-values were 0.022 and 0.083, respectively.

The results of these analyses indicate that BALSAMICO has an advantage over other state-of-the-art methods, SpNMF, when investigating the relationship between multiple explanatory variables and bacterial communities.

## Conclusions

We proposed a novel hierarchical Bayesian model to discover the underlying microbial community structures and the associations between microbiota and their environmental factors based on microbial metagenomic data. One of the most important features of our model is to decompose the contribution matrix into observed environmental factors and their coefficients. The parameters for this model were estimated using variational Bayesian inference, as described in “Methods”. In terms of computation, this parameter-estimation procedure offers two advantages over existing methods. First, in an algorithm that uses Gibbs sampling, the computational cost is large due to the large number of samples required. The Gibbs sampler requires directly sampling latent variables *s*_*n*,*l*,*k*_. Therefore, the per-iteration computational complexity of the Gibbs sampling procedure is $\mathcal {O}(NK)$. By contrast, our procedure involves a matrix operation that substitutes for this requirement, helping to reduce the computational cost. The variational inference can directly update sufficient statistics $\sum _{k} s_{n,l,k}$ and $\sum _{n} s_{n,l,k}$ (see Supplemental materials). This reduced practical calculation time. In the analysis of Zeller’s data, with *L*=7, the calculation time in our Mac book (processor; 3.5 GHz Intel Core i7 and memory; 16 GB 2133 MHz LPDDR3) was 9.378 seconds.

Second, our procedure involves hyper-parameter tuning. The parameters of the gamma prior distribution are estimated from the data. The parameters of the Dirichlet prior distribution can be non-informative, and the number of communities *L* can be selected by cross-validation.

The results of our simulations suggest that the estimators of the effects of environmental factors ***V*** are consistent. Generally, other NMF methods lack consistency because they may not have a unique solution [16]. Indeed, the consistency of our method increases the reproducibility of the analysis. Moreover, the credible intervals of coefficient ***V*** are easily computed and help to identify notable bacteria.

From the perspective of data analysis, BALSAMICO has useful properties. Using the Dirichlet prior distribution, the excitation matrix ***H*** is easily interpreted as a relative abundance of species in communities. As shown in Fig. 13, *h*_*l*,*k*_ obtains a value that is often close to zero. This property thus expresses data sparsity. Furthermore, the Poisson observation model may be applicable to other count data (for example, gene expression data). The hierarchical structure of our model allows it to capture (*i*) dependencies between environmental factors and the community structure (represented by coefficient ***V***), and (*ii*) the individual differences in microbial composition (represented by the contribution matrix ***W***). Thus, BALSAMICO can be used to find latent relationships between bacteria. As discussed in “Results,” BALSAMICO’s findings from real data are supported by previous studies. This demonstrates that BALSAMICO is effective at knowledge discovery.

This research has possibilities for expansion and may provide positive contributions to future studies. In many situations, microbiome data is obtained as time series with repeated measurements for each sample. To handle the time series data, our model could be expanded so the contribution matrix ***W*** is extended from a matrix to a tensor. This facilitates the analysis of time-varying bacterial composition during the progression of a disease. Furthermore, although this research was limited to the study of the human microbiome, BALSAMICO will prove useful to other studies seeking to find relationships between various microbiomes and environmental factors. This will allow for a better understanding of the cause of disease and how disease is impacted by the microbiome environment.

## Availability and requirements

- Project name: BALSAMICO
- Project home page: https://github.com/abikoushi/BALSAMICO
- Operating system: Platform independent
- Programming language: R
- Other requirements: R 4.0.3 or higher
- License: GNU GPL
- Any restrictions to use by non-academics: none

## Supplementary Information

**Additional file 1** Supplemental methods. Details of variational inference.

## Data Availability

BALSAMICO is implemented with R and is available from GitHub (https://github.com/abikoushi/BALSAMICO). All datasets used in this study have been previously published. Zeller’s data is available in the R package “curatedMetagenomicData” (https://github.com/waldronlab/curatedMetagenomicData). Gajer’s data is available in the [28] (https://stm.sciencemag.org/content/4/132/132ra52). David’s data is available in the R package “themetagenomics” (https://github.com/EESI/themetagenomics).

## Abbreviations

CP: coverage probability
CRC: Colorectal cancer
HMP: Human Microbiome Project
MetaHIT: Metagenomics and the Human Intestinal Tract
NMF: Non-negative matrix factorization
OTU: Operational taxonomic unit
RMSE: Root mean squared error
SD: Standard deviation
